# DNA Analysis Indicates That Asian Elephants Are Native to Borneo and Are Therefore a High Priority for Conservation

**DOI:** 10.1371/journal.pbio.0000006

**Published:** 2003-08-18

**Authors:** Prithiviraj Fernando, T. N. C Vidya, John Payne, Michael Stuewe, Geoffrey Davison, Raymond J Alfred, Patrick Andau, Edwin Bosi, Annelisa Kilbourn, Don J Melnick

**Affiliations:** **1**Center for Environmental Research and Conservation, Columbia UniversityNew York, New YorkUnited States of America; **2**Department of Ecology, Evolution, and Environmental Biology, Columbia UniversityNew York, New YorkUnited States of America; **3**Center for Ecological Sciences, Indian Institute of ScienceBangaloreIndia; **4**World Wide Fund for Nature–MalaysiaKota Kinabalu, SabahMalaysia; **5**Asian Rhino and Elephant Action Strategy Programme, World Wildlife FundWashington, District of ColumbiaUnited States of America; **6**Sabah Wildlife DepartmentKota Kinabalu, SabahMalaysia; **7**Field Veterinary Program, Wildlife Conservation SocietyBronx, New YorkUnited States of America

## Abstract

The origin of Borneo's elephants is controversial. Two competing hypotheses argue that they are either indigenous, tracing back to the Pleistocene, or were introduced, descending from elephants imported in the 16th–18th centuries. Taxonomically, they have either been classified as a unique subspecies or placed under the Indian or Sumatran subspecies. If shown to be a unique indigenous population, this would extend the natural species range of the Asian elephant by 1300 km, and therefore Borneo elephants would have much greater conservation importance than if they were a feral population. We compared DNA of Borneo elephants to that of elephants from across the range of the Asian elephant, using a fragment of mitochondrial DNA, including part of the hypervariable d-loop, and five autosomal microsatellite loci. We find that Borneo's elephants are genetically distinct, with molecular divergence indicative of a Pleistocene colonisation of Borneo and subsequent isolation. We reject the hypothesis that Borneo's elephants were introduced. The genetic divergence of Borneo elephants warrants their recognition as a separate evolutionary significant unit. Thus, interbreeding Borneo elephants with those from other populations would be contraindicated in ex situ conservation, and their genetic distinctiveness makes them one of the highest priority populations for Asian elephant conservation.

## Abstract 2

Comparison between DNA sequences of Borneo elephants with those of other Asian elephants settles a longstanding dispute about the origins of these endangered animals

## Introduction

**Figure 1:**
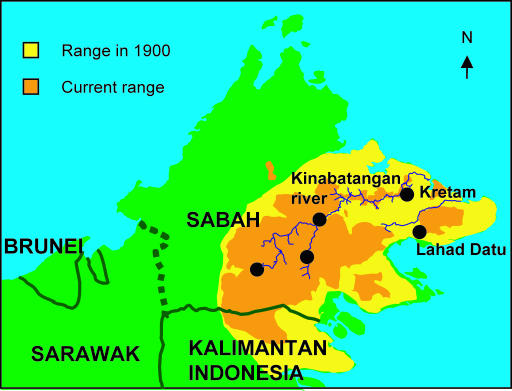
Asian Elephant Range and Sampling Locations in Borneo Solid lines demarcate country borders and the dotted line the boundary between the Malaysian states of Sabah and Sarawak. Black dots indicate areas of sample collection.

Elephants have a very limited distribution in Borneo, being restricted to approximately 5% of the island in the extreme northeast (Figure 1). There are no historical records of elephants outside of this range. Fossil evidence for the prehistoric presence of elephants on Borneo is limited to a single specimen of a tooth from a cave in Brunei (Hooijer 1972).

Popular belief holds that elephants presented to the Sultan of Sulu in 1750 by the East India Trading Company and subsequently transported to Borneo founded the current population (Harrisson and Harrisson 1971; Medway 1977). These animals presumably originated in India (Shoshani and Eisenberg 1982), where company operations and trade in domesticated elephants were centred. Alternatively, considering the geographic proximity to Borneo, the elephant trade that flourished in Sumatra and peninsular Malaysia during the 16th–18th centuries (Andaya 1979; Marsden 1986[1811]) may have been the source. Thus, if elephants were introduced to Borneo, the source population could have been India, Sumatra, or peninsular Malaysia, and as a feral population, Borneo's elephants would have low conservation importance.

**Figure 2:**
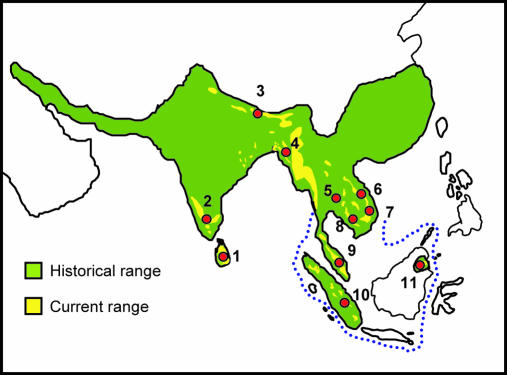
Asian Elephant Range and Sampling Locations Central sampling locations denote the countries sampled and represent a number of actual sampling locations within each country. 1. Sri Lanka, 2. India, 3. Bhutan, 4. Bangladesh, 5. Thailand, 6. Laos, 7. Vietnam, 8. Cambodia, 9. Peninsular Malaysia, 10. Sumatra (Indonesia) 11. Borneo (Sabah–Malaysia).

Conversely, if elephants occurred naturally on Borneo, they would have colonised the island during Pleistocene glaciations, when much of the Sunda shelf was exposed (Figure 2) and the western Indo-Malayan archipelago formed a single landmass designated as Sundaland (MacKinnon et al. 1996). Thus, the isolation of Borneo's elephants from other conspecific populations would minimally date from the last glacial maximum, 18,000 years ago, when land bridges last linked the Sunda Islands and the mainland (MacKinnon et al. 1996). If Borneo's elephants are of indigenous origin, this would push the natural range of Asian elephants 1300 km to the east, and as a unique population at an extreme of the species' range, Borneo elephants' in situ conservation would be a priority and ex situ cross-breeding with other populations would be contraindicated.

Initially, Borneo elephants were classified as a unique subspecies (Elephas maximus borneensis) based on morphological differences from other populations (Deraniyagala 1950, 1955). Subsequently, they were subsumed under the Indian Elephas maximus indicus (Shoshani and Eisenberg 1982) or the Sumatran Elephas maximus sumatrensis (Medway 1977) subspecies, based on an assumption of their introduction to the region or on the reasoning that morphological divergence was insufficient to warrant separate status. While unique subspecific status would highlight their conservation importance, evaluation of their status in terms of evolutionary significant units (ESUs) and management units (MUs) (Ryder 1986; Moritz 1994) would be more relevant to conservation management.

## Results

**Figure 3:**
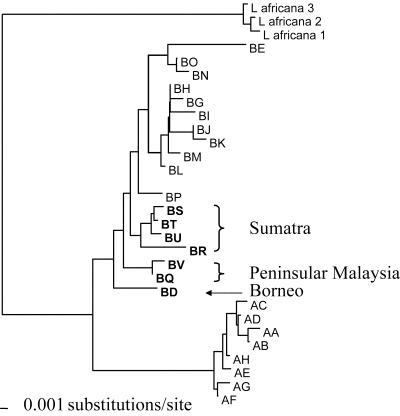
A Neighbour-Joining Phylogram of Asian Elephant Haplotypes Rooted with an African Elephant Out-Group Sunda Region haplotypes are in bold.

**Figure 4:**
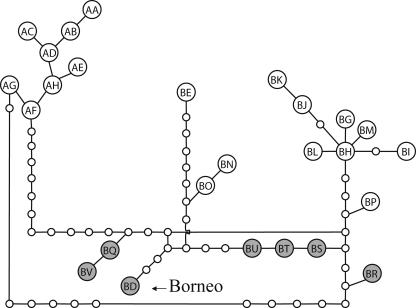
Network of Asian Elephant Haplotypes Based on Statistical Parsimony Grey circles with letters denote haplotypes unique to the Sunda region (BD: Borneo; BQ, BV: peninsular Malaysia; BR, BS, BT, BU: Sumatra). White circles with letters denote haplotypes found in mainland Asia (excluding peninsular Malaysia) and Sri Lanka. The small open circles denote hypothetical haplotypes. Haplotypes beginning with the letters A and B belong to the two clades α and β, respectively.

We PCR-amplified and sequenced a 630 bp fragment of mitochondrial DNA (mtDNA), including the hypervariable left domain of the d-loop (Fernando et al. 2000), from 20 Borneo elephants and compared them with 317 sequences we generated for elephants across ten of the 13 Asian elephant range states (Figure 2). Asian elephant haplotypes segregated into two distinct clades, α and β (Fernando et al. 2000). All ‘Sundaland’ (peninsular Malaysia, Sumatra, and Borneo) haplotypes fell in clade β, while α and β clades were observed in Sri Lanka and mainland populations (Figures 3 and 4). The Borneo population was fixed for the unique β-haplotype BD. Similar tree topologies were obtained by maximum parsimony, neighbour joining, and maximum-likelihood methods of phylogenetic analyses, with some minor rearrangements of the terminal branches. In all trees, Bornean and other haplotypes unique to ‘Sundaland' (Borneo: BD; peninsular Malaysia: BQ, BV; Sumatra: BS, BU, BT, BR) occupied basal positions in the β-clade phylogeny (Figure 3) and were derived from internal nodes in a parsimony network of haplotypes (Figure 4). Uncorrected *p* distances between the Borneo haplotype and other β-haplotypes ranged from 0.012 (haplotypes BQ, BP, BO, BS, BU) to 0.020 (haplotype BE), with a mean of 0.014. Assuming a nucleotide substitution rate of 3.5% per million years for the elephant mtDNA d-loop (Fleischer et al. 2001), the observed genetic distance indicates divergence of the Borneo haplotype BD and its closest relative from a common ancestor approximately 300,000 years ago. Owing to stochastic coalescent processes, the use of a single gene to infer population parameters is prone to error. Despite any such error, the magnitude of the genetic difference between Borneo and other Asian elephant haplotypes is such that it indisputably excludes divergence since introduction; the observed divergence is so great that even if there was some error it would not have any influence on the conclusion that places the Borneo haplotype in a timeframe supporting a Pleistocene colonisation rather than introduction by humans.

We also genotyped 15 Borneo elephants for five polymorphic autosomal microsatellite loci (Nyakaana and Arctander 1998; Fernando et al. 2001) and compared them to 136 five-locus genotypes we generated for Asian elephants from nine range states. Tests of Hardy–Weinberg equilibrium and linkage disequilibrium in all populations indicated simple Mendelian inheritance of five unlinked, selectively neutral loci. The total number of alleles per locus across populations in the Asian elephant ranged from 2.0 (*EMX-2*) to 11.0 (*LafMS03*) (*x¯*, SE = 4.60, 1.51); the average number of alleles across loci, per population (excluding Borneo), from 2.0 (Sumatra) to 3.6 (Sri Lanka) (*x¯*, SE = 2.93, 0.155); the observed heterozygosity H_0_ across all populations (excluding Borneo) from 0.38 (*EMX-4*) to 0.63 (*LafMS03*) (*x¯*, SE = 0.44, 0.041); and gene diversity from 0.39 (*EMX-4*) to 0.69 (*LafMS03*) (*x¯*, SE = 0.47, 0.050). Comparatively, all indices demonstrated very low genetic diversity in the Borneo population: proportion of polymorphic loci, 0.4; number of alleles per locus, 1–2 (*x¯*, SE = 1.40, 0.219); gene diversity, 0–0.13 (*x¯*, SE = 0.04, 0.024); heterozygosity H_0_ = 0–0.07 (*x¯*, SE = 0.01, 0.013). The number of alleles, observed heterozygosity, and gene diversity, averaged across Asian elephant populations, were all higher than those in Borneo, at all loci (Table 1). Similarly, in all populations, the number of alleles and observed heterozygosity, averaged across loci, were higher than in Borneo (Table 2). Five unique genotypes were identified in the 15 Borneo elephants sampled. In tests of population subdivision, all pairwise comparisons between Borneo and other populations demonstrated highly significant differentiation, *F_ST_* 0.32–0.63 (*x¯*, SE = 0.44, 0.034) (Table 3). In tests of a recent bottleneck, no heterozygote excess (Maruyama and Fuerst 1985) or mode-shift distortion of allele frequency distributions (Luikart et al. 1998a), characteristic of a recent bottleneck, was observed in the Borneo population. In assignment tests indicating the distinctness of a population's genotypes, all five Borneo genotypes were assigned with maximum likelihood to Borneo (likelihoods ranging from 0.004 to 0.80, *x¯*, SE = 0.51, 0.175), and maximum-likelihood ratios of the most-likely (Borneo) to the next-most-likely population ranged from 2.97 to 48.20 (*x¯*, SE = 25.02, 8.795). Borneo was significantly more likely to be the source than any other population for all five genotypes, since each of the assignment likelihoods to Borneo fell outside the upper end of the corresponding distribution of assignment likelihoods to the other populations. Assignment likelihoods to the putative Indian, Sumatran, and peninsular Malaysian source populations were very small (India: 0–0.0004, *x¯*, SE = 0.000126, 0.000065; Sumatra: 0–0.0355, *x¯*, SE = 0.007146, 0.006336; peninsular Malaysia: 0.0003–0.1195, *x¯*, SE = 0.0301, 0.0201), indicating that Borneo's genotypes were highly unlikely to have originated from any of these populations.

**Table 1:**
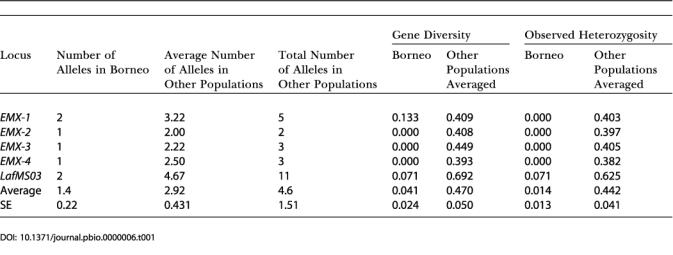
Comparison of Measures of Genetic Variation at Individual Loci in Borneo with Those of the Other Populations

**Table 2:**
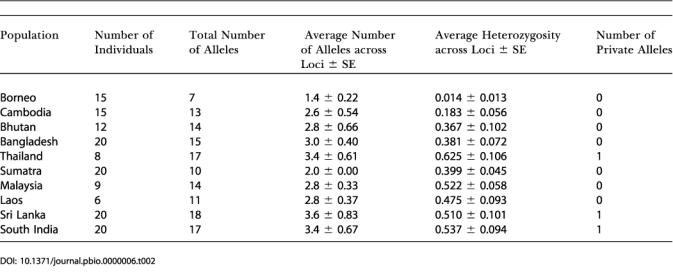
Measures of Genetic Variation Using Five Loci, in Asian Elephant Populations from across the Range

**Table 3:**
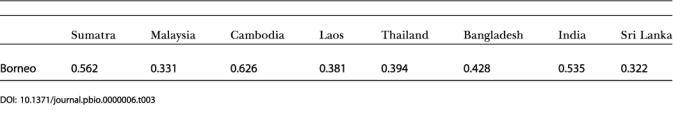
F*_ST_* Values in Pairwise Comparison of Borneo with Other Populations

## Discussion

mtDNA evidence supports an indigenous hypothesis in three ways. First, this hypothesis assumes an ancient, independent evolution of Borneo's elephants, resulting in the unique, divergent Borneo haplotype(s), as we observed. Conversely, the introduction hypothesis assumes an introduction at 500 years ago or less, which approximates zero time on a scale of mtDNA d-loop evolution, and hence requires Borneo and source population haplotypes to be identical. This was not observed. Second, the estimated divergence time between the Borneo haplotype and other Asian elephant haplotypes is concordant with a mid- to late-Pleistocene isolation of elephants on Borneo and the vicariant history of the island (MacKinnon et al. 1996). Third, all observed ‘Sundaland' haplotypes, including Borneo's, were of the β clade, had basal relationships to that clade in a phylogenetic tree, and were independently derived from internal nodes in a haplotype network, suggesting an ancient isolation of these lineages on Borneo, Sumatra, and peninsular Malaysia. Thus, the Borneo haplotype fits a pattern of distribution and relatedness to other ‘Sundaland' haplotypes that is congruent with an ancient colonisation of the Sunda region by β clade and subsequent allopatric divergence of populations on its larger landmasses.

Microsatellite data also support the indigenous hypothesis. If the Borneo population originated from animals introduced in the 16th–18th centuries, it would have reached its mid-20th-century size of approximately 2,000 individuals (deSilva 1968) in fewer than 30 generations, assuming an Asian elephant generation time of 15–20 years (Sukumar 1989). Thus, the Borneo population would have experienced a rapid demographic expansion after the ‘recent’ bottleneck caused by the founder-event of introduction. We did not observe a heterozygote excess or a mode-shift distortion in allele frequency distribution in the Borneo population, suggesting that the population did not undergo a recent bottleneck and hence did not arise from a few introduced animals. However, this result by itself is not conclusive, since with a sample size of 15 and five loci, the test for heterozygosity excess has low power and bottlenecks may not be detected (Luikart et al. 1998b). We observed extremely low genetic diversity at Borneo elephant microsatellite loci, including fixation at three of the five loci. Sequential founder-events or persistent small population size, as would be expected in a small population isolated since the Pleistocene, would lead to substantial loss of genetic variation (Nei et al. 1975) and hence is consistent with the data. Successful founding of a population by a very few individuals from a single introduction could also result in a severe bottleneck. However, given the adversities faced by translocated elephants (Fernando 1997) and the importance of social structure in the reproduction and survival of elephants (Fernando and Lande 2000; McComb et al. 2001), such an explanation is unlikely.

In the assignment tests, all five Borneo genotypes, which included free-ranging as well as captive animals, were assigned to Borneo with significantly higher likelihoods than to other populations and with extremely low likelihoods to the putative source populations. An introduced population may be highly divergent from the source population in terms of *F* statistics (Williams et al. 2002) due to allelic loss from founder-events. However, the probability of loss for a particular allele is inversely proportional to its frequency in the founder and hence the source population. Thus, genotypes in an introduced population would retain a high likelihood of assignment to the source population, enabling its identification from among a number of candidate populations. Therefore, the assignment tests strongly suggest that the Borneo elephants were not derived from another population in the recent past.

Thus, microsatellite data strongly suggest a Pleistocene colonisation, independent evolution through a long period of isolation, and long-term small population size for the Borneo population. It strongly rejects a recent origin from any of the putative source populations.

Mitochondrial and microsatellite analyses indicate that Borneo's elephants are indigenous to Borneo, have undergone independent evolution since a Pleistocene colonisation, and are not descended from animals introduced by humans. The evolutionary history of Borneo's elephants warrants their recognition as a separate ESU (Moritz 1994). Thus, they should not be cross-bred with other Asian elephants in ex situ management. The genetic distinctiveness and evolutionary history of Borneo elephants support their recognition as a unique subspecies. However, one of the reasons E. maximus borneensis was subsumed under E. m. indicus and E. m. sumatrensis was the inadequacy of the original description of E. m. borneensis in terms of the morphological characters assessed and sample size. Therefore, we suggest that a formal reinstatement of the E. m. borneensis taxa await a detailed morphological analysis of Borneo elephants and their comparison with other populations.

While Borneo's elephants appear to be genetically depauperate, through a long history of isolation and inbreeding, they may have purged deleterious recessive alleles from their genome and decreased their genetic load, thus becoming less susceptible to inbreeding depression. We recommend research on reproductive rates, juvenile survival, and other indicators of detrimental effects of inbreeding such as sperm deformities, sperm mobility, and genetic diversity at MHC loci. While increasing genetic diversity by introducing a small number of elephants from other populations (Whitehouse and Harley 2001) may have to be considered if deleterious inbreeding effects are evident, in the absence of such findings Borneo's elephants should be managed separately from other Asian elephants.

## Materials and Methods

### 

#### Samples.

Samples consisted of dung from free-ranging and dung or blood from captive elephants. Sample collection, storage, and DNA extraction followed published protocols (Fernando et al. 2000, 2003). For mitochondrial and microsatellite analysis, respectively, 20 and 15 samples from Borneo (nine blood samples from elephants captured for management purposes—eight from the Kretam area and one individual originating from around Lahad Datu—and the rest from dung samples from free-ranging elephants collected during a survey of the Kinabatangan watershed) were compared with 317 and 136 samples from across the current Asian elephant range, Sri Lanka (n = 81, 20), India (n = 81, 20), Bhutan (n = 13, 13), Bangladesh (n = 30, 20), Thailand (n = 8, 8), Cambodia (n = 30, 20), Vietnam (n = 5, 0), Laos (n = 20, 6), Indonesia (Sumatra) (n = 40, 20), and peninsular Malaysia (n = 9, 9). Vietnam was excluded from the microsatellite analysis owing to nonamplification of a number of samples.

#### mtDNA amplification and sequencing.

Approximately 630 bp of mtDNA, including the left domain of the d-loop, were amplified using published primers (Fernando et al. 2000). PCR products were sequenced in both directions, using internal sequencing primers MDLseq-1 (CCTACAYCATTATYGGCCAAA) and MDLseq-2 (AGAAGAGGGACACGAAGATGG), and resolved in 4% polyacrylamide gels in an ABI 377 automated sequencer (Perkin-Elmer, Wellesley, Massachusetts, United States).

#### mtDNA phylogenetic analysis.

We used 600 bp of the amplified segment in the analysis. Sequences were aligned and edited using SEQUENCHER version 3.1.1 (GeneCodes Corporation, Ann Arbor, Michigan, United States). Sequences were deposited in GenBank (accession numbers AY245538 and AY245802 to AY245827). Phylogenetic analyses were conducted using PAUP* version 4.0 (Swofford 1998). Three African elephant (Loxodonta africana) sequences from zoo animals in the United States were used as an out-group. Genetic distances among sequences were calculated using uncorrected *p* distances. Maximum-parsimony analysis was conducted using a heuristic search with random stepwise addition of taxa, tree bisection/reconnection branch swapping, and equal weighting; neighbour joining, with Kimura two-parameter distances; and maximum likelihood, using empirical base frequencies and estimated values for the shape parameter for among-site rate variation and transition/transversion ratios. A network of haplotypes was created using statistical parsimony in the software TCS version 1.13 (Clement et al. 2001).

#### Microsatellite amplification.

Samples were screened with five published microsatellite loci, *EMX-1* to *EMX-4* (Fernando et al. 2001) and *LafMS03* (Nyakaana and Arctander 1998). Forward primers were fluorescent labelled (FAM, HEX, or TET), samples were amplified in 12.5 μl volumes with relevant cycling profiles (Fernando et al. 2001), and 1 μl of PCR product was mixed with 0.2 μl of loading-dye and 0.5 μl of Tamra 500 size standard (Applied Biosystems, Foster City, California, United States) and was resolved in 4% polyacrylamide gels in an ABI 377 automated sequencer. Alleles were scored using GENESCAN software (Applied Biosystems) and published guidelines (Fernando et al. 2003).

#### Microsatellite data analysis.

Deviations from Hardy–Weinberg equilibrium for each locus and population were tested using the exact Hardy–Weinberg test as implemented in GENEPOP 3.2 (Raymond and Rousset 1995), with the complete enumeration method (Louis and Dempster 1987) for loci with fewer than four alleles and with the Markov chain method (Guo and Thompson 1992) (dememorization: 1000; batches: 100; iterations per batch: 1000) for loci with more than four alleles. GENEPOP was also used to test for linkage disequilibrium between loci, using the Markov chain method. Population differentiation was tested with estimates of Wright's fixation index (Weir and Cockerham 1984), *F_ST_*, using the program Arlequin version 2 (Schneider et al. 2000).

Evidence for a recent bottleneck in the Borneo population in terms of a heterozygote excess (Cornuet and Luikart 1996) or a mode-shift distortion in allele frequencies (Luikart et al. 1998a) was conducted using the program BOTTLENECK version 1.2.02 (Piry et al. 1997) and a graphical method (Luikart et al. 1998a).

Assignment tests were performed using WHICHRUN version 4.1 (Banks and Eichert 2000). Assuming Hardy–Weinberg equilibrium in each baseline population and linkage equilibrium between loci, the likelihood that an individual originates from a particular population is the Hardy–Weinberg frequency of the individual's genotype at that locus, in that population. This likelihood was multiplied across loci to obtain a multilocus assignment likelihood of the test individual to each population, and the population with the highest value was identified as the ‘most-likely’ source population. To test for statistical significance of the most-likely source population, this assignment likelihood was compared with the distribution of assignment likelihoods of the other populations. Maximum-likelihood ratios were calculated as the ratio between the likelihood of assignment to the most-likely population to that for a particular population.

## Supporting Information

### 

#### Accession Numbers

The GenBank accession numbers for the sequences reported in this paper are AY245538 and AY245802 to AY245827.

## Abbreviations

ESU: evolutionary significant unit
mtDNA: mitochondrial DNA
MU: management unit.
